# Study of trace metal contamination and ecological risk assessment in the sediments of a tropical river estuary, Southwestern India

**DOI:** 10.1007/s10661-021-09728-1

**Published:** 2022-01-14

**Authors:** D’Souza Nishitha, Vadakkeveedu Narayan Amrish, Kumar Arun, Anish Kumar Warrier, Harikripa Narayana Udayashankar, Keshava Balakrishna

**Affiliations:** Department of Civil Engineering, Manipal Institute of Technology, Manipal Academy of Higher Education, Manipal, India

**Keywords:** Estuarine sediments, Metals, Particle size distribution, Pollution, Risk index, Southwest India

## Abstract

The present study aims to assess the extent of trace metal pollution in the sediments of Sita-Swarna estuary, west coast of India, and investigate their possible ecological risk on the aquatic environment. The sediment cores were analyzed for sand, silt, clay, organic carbon, and trace metals (Al, Fe, Mn, As, Cd, Co, Zn, Pb, Ni, Cr, and Cu) at 2-cm intervals. The study revealed that sediments have deposited in relatively violent to very violent hydrodynamic energy conditions. Factor analysis indicated that the metal distribution is mainly controlled by Fe–Mn oxyhydroxides and organic carbon. Further, the geochemical approach, pollution indices, and statistical evaluation revealed moderate pollution in the catchment. From an ecotoxicological perspective, the estimated risk index (RI) value was found to less than 150, indicating low risk for aquatic life. Thus, this baseline study would help to adopt strategies in pollution control and protect the fragile marine environment.

## Introduction

Civilization is often associated with rivers and coastal ecosystems from the past to the present. Specifically, estuaries are of global importance because they are viewed as highly productive zones and sustain unique plant and animal populations (Gredilla et al., 2015). However, with the rising pollution of the fluvial and coastal ecosystems, the current scenario alarms humanity and marine life (Bingöl et al., 2013; Fernandes et al., 2008; Tavakoly Sany et al., 2014; Zahra et al., 2014). Due to their non-degradability and ability to bioaccumulate in the food chain, metal pollution has become a global problem and gaining the prime attention of the decision-makers (Bastakoti et al., 2019; Ergül et al., 2013; Prajith et al., 2016; Xavier et al., 2020). Hence investigation of biogeochemistry of rivers and estuaries provides insights to the trace metal toxicity and its bioavailability for the well-being of aquatic biota. Furthermore, estuaries are very dynamic environments that can significantly alter the amount of organic and inorganic pollutants entering the coastal environment before depositing into the deep sea and mixing with the marine sediments. Changes in the ionic strength, pH, velocity of the water, and the ratios of divalent cations result in the flocculation of finer particles. The finer ones eventually end up in the bottom sediments, which are known as contaminant sinks. Thus, sediments are the most significant pools for trace metals (Zhang et al., 2015; Zhao et al., 2017).

On the other hand, contaminated sediments will not remain at the bottom perpetually; varying hydrodynamic conditions and geochemical processes lead to resuspension of contaminants between sediments and water column, which can adversely affect the aquatic life (Atkinson et al., 2007; Hu et al., 2013). There are many factors such as grain size (Fernandes & Nayak, 2016; Mokwe-Ozonzeadi et al., 2019; Nasnodkar & Nayak, 2015), organic matter (Ghosh et al., 2016), mangroves (Bastakoti et al., 2019; Xavier et al., 2020), and hydrodynamic conditions (Dessai et al., 2009; Liu et al., 2019) in regulating the deposition and distribution of trace metals. Also, sediments are significant in archiving past environmental and climate-related changes (Ahmed et al., 2018; Alharbi et al., 2019); thus, it is possible to predict the past contamination vis-à-vis the present situation. Timely investigation of metal pollution in the rivers, estuaries, and the adjacent coastal environments will determine the magnitude of pollution levels (Cyriac et al., 2021; Mitra et al., 2018).

Though tropical rivers account for greater than 60% of the water and sediment discharge to the world oceans (Gaillardet et al., 1995), very few studies exist on the dissolved trace metal behavior in the estuarine environment of tropics (Alagarsamy, 2006; Cyriac et al., 2021; Satapathy & Panda, 2015). Specific to Sita-Swarna rivers, only two studies are documented as-on-date (Nishitha et al., 2019; Tripti et al., 2013). Hence to fill the gap in the literature, this study has been taken up in the estuarine environment of Sita-Swarna. With this overview, we have attempted an in-depth study to understand the factors controlling the distribution pattern of the metals in the Swarna estuary. The banks of the Swarna are densely populated and have a major fishing harbor (Malpe) of Karnataka coast. In the future, this study can be used to compare the varying heavy metal pollution with time, in the catchment. Policy makers will benefit from the data reported, for framing future pollution mitigation strategies.

The main objective of the work is (i) to study the down core variations of the metals, (ii) to study the influence of grain size in the deposition of metals, and (iii) to assess the sediment quality using various geochemical indices.

## Study area

**Fig. 1:**
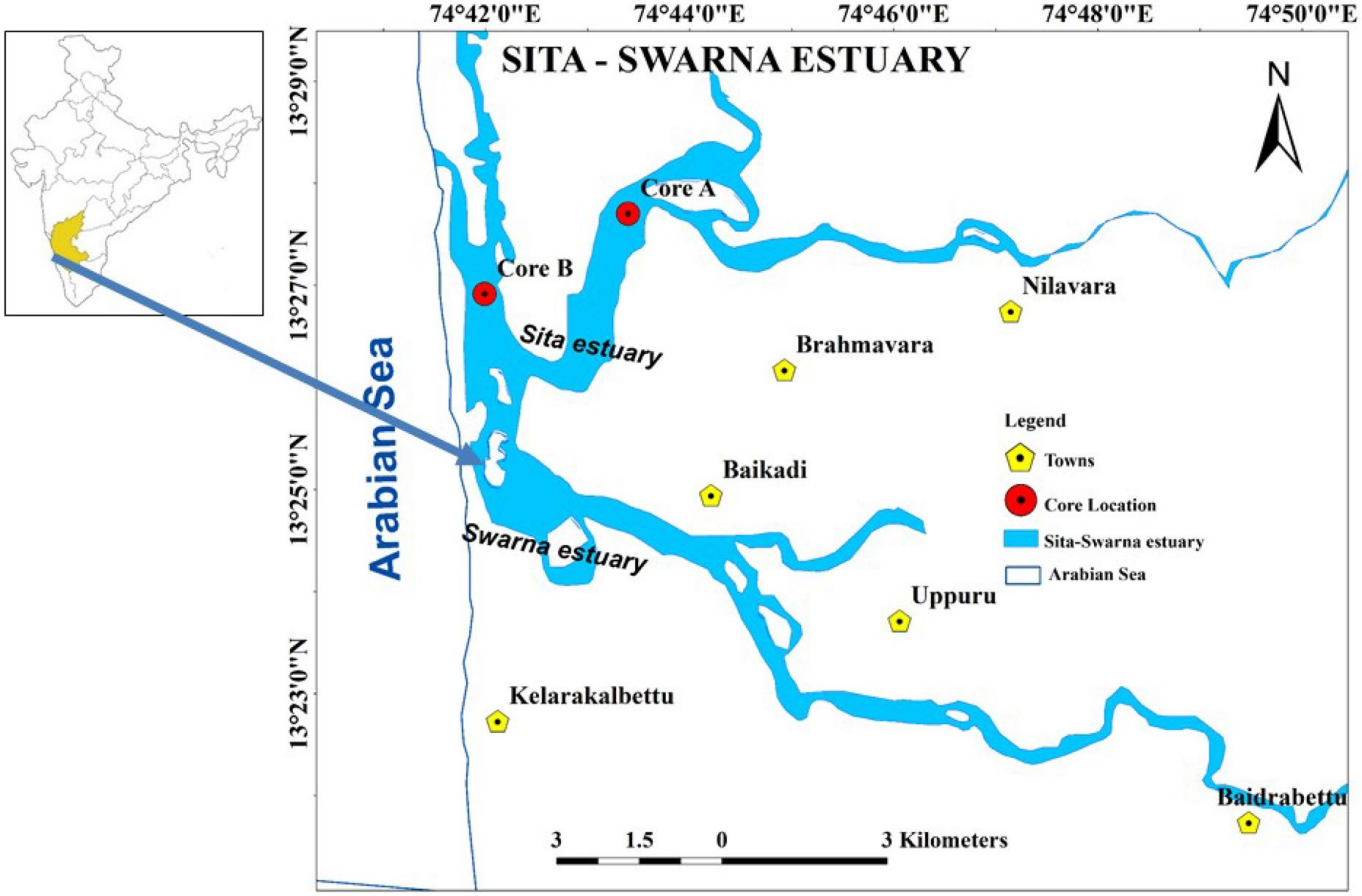
Map showing the core locations in the Sita-Swarna estuary (modified map from Nishitha et al. (2021)

The study area (Fig. 1) lies between 13°23′30″N and 13°28′30″N latitudes and between 74°41′55″E and 74°45′00″E longitudes. The estuary remains freshwater dominated during the southwest monsoon (June to September) and saline during the rest of the year. The water levels during the neap period range from 0.5 to 1.0 m (low) and from 1.0 to 1.5 m (high), respectively. The waves along the coast are low during pre-monsoon and post-monsoon, with a wave height of ~ 1.2 m and 1.4 m, respectively. In contrast, it reaches a maximum of 4 m during the southwest monsoon in the western direction (Avinash et al., 2012).

## Materials and methods

### Raising and subsampling of sediment core

Two shallow cores were collected from the Sita-Swarna estuary, one upstream near the mangrove vegetation (Core A — 70 cm) and one downstream near the mudflat (Core B — 76 cm) (Fig. 1). The cores were collected by manually pushing a PVC pipe into the sediment bed during the low-tide conditions. The cores were labeled, packed, and stored in cold storage (at 4 °C) for further analysis. Later, both the cores were subsampled at 2-cm intervals using a plastic knife to avoid metal contamination. Subsamples were transferred to zip lock polyethylene bags and stored for further analysis. Visually, core A was grey in color and core B brownish.

### Digestion of sediments for metal analysis

The wet sediment samples were oven-dried at a temperature of less than 60 °C. Later, the dried samples were sieved to obtain the fine fraction (< 63 µm). Approximately 0.1 g of sediment was weighed and transferred to Teflon beakers. An acid mixture of 2 mL of Merck Suprapur® HF (40%), 5 mL of HNO_3_ (65%), and 2 mL of HCl (35%) was added to the sediment sample (Suja et al., 2017). Further, the samples were transferred onto a hot plate and kept for digestion. Initially, the temperature was maintained at 90 °C for 2 h and later decreased to 60 °C until the samples evaporated to dryness. The residue was dissolved in 0.1 N HNO_3_. This was repeated 2 to 3 times to remove excess HF present in the samples. Finally, the residue was made up to 25 mL using 0.1 N HNO_3_ and transferred to cleaned PP bottles. The digested samples were preserved in cold storage at 4 °C. Digestion was performed in a fume hood; extreme care was taken to avoid any metal contamination. Further, samples were analyzed for metals using ICP-OES Thermo fisher ICAP 7000 series available at the Department of Civil Engineering, Manipal Institute of Technology. The repeatability of the samples was checked by analyzing the duplicates and known concentration standards. One control standard was placed in the sample analysis sequence for every ten samples. The accuracy of the measurements was ensured by running a certified estuarine reference standard, BCR®-667. The certified standard was digested and run along with the samples. The accuracy and precision of the measurements were < 6% and 5%, respectively. Further multivariate statistical analysis was performed using the Statistical Package for the Social Sciences (SPSS)—v.21. Also, factor analysis (FA) with principal component extraction and Kaiser normalized varimax rotation was performed on the data sets.

### Grain size analysis

Approximately 10 g of oven-dried (at 60 °C) sediment samples were used for particle size analysis. The organic matter and carbonates were removed by treating the sample with 30% hydrogen peroxide and 10% glacial acetic acid (Warrier et al., 2016). Later wet sieving was done using ASTM sieve size 230 (62-µm pore diameter) to separate the sand fraction in the sample. After removing the sand fraction, the silt and clay fraction mixture was transferred to a graduated cylinder. To prevent the flocculation of the particles, the deflocculating agent 20 mL of sodium hexametaphosphate (Calgon) was added. By using the conventional method of pipette analysis, silt + clay was determined. The cylinder containing clay and silt was stirred vigorously for about 1 min using a long PVC pipe of 25-mm diameter. After 20 s of stirring, 20 mL of sample was pipetted out from 20-cm depth. The collected suspensions were dried in an oven (< 100 °C). The duplicate sample was collected before 40 s and analyzed for silt and clay fraction using a particle size analyzer available at the Geological Survey of India (GSI), Mangalore. The weight of the silt fraction was multiplied by a factor of 50, and 1 g was subtracted from the value obtained to account for the Calgon solution that was added before. A similar procedure was followed for clay fractions. Dry sieving was carried out at half-phi intervals for the coarser particles retained on the + 230 sieve (5, 10, 18, 35, 60, 120, + 230, and Pan). The sieves are assembled with coarser mesh at the top, followed by finer mesh and agitated using a mechanical shaker for 5 min. Sieved individual fractions were weighed, and weight percentages calculated. Further, GStat, an in-house software available at GSI, Mangalore, and the grain size distribution statistics were estimated.

### Contamination assessment methods

Sediment assessment can be done using various methods. The most used ones are the geoaccumulation index and enrichment factor. The *I*_geo_ was calculated to determine the degree of pollution in the sediments (Muller, 1969). The estimated concentrations were compared with the geochemical background values, i.e., average shale values given by Turekian and Wedopohl (1961). The results were interpreted using the following equation:

$$I_\text{geo}=(\log_2\times (C_n/1.5\times B_n))$$

where *C*_*n*_ is the element of interest and *B*_*n*_ is the background value. A factor of 1.5 was used to account for the lithological variations for geochemical background values. Similarly, a normalized enrichment factor (Ergin et al., 1991) was applied to differentiate the source of origin from anthropogenic and natural means. This involved normalizing the sediments with conservative elements concerning reference elements such as Al, Fe, Ti, and Mn. In the current study, normalization was done with metal Al using the average shale value given by Turekian and Wedopohl (1961). The following equation calculated the Enrichment Factor (EF):

$$\text{EF}=\frac{(C/R)\text{ Sample}}{(C/R) \text{ World average shale}}$$

where (*C*/*R*)_**Sample**_ is the ratio of the concentration of element of interest (*C*) to the reference element (*R*) in the sediment samples and (*C*/*R*)_**average shale value**_ is the ratio of the concentration of element of interest (*C*) to the reference element (*R*) in the geochemical background.

Further to evaluate the potential risk of trace metals on the ecosystem, ecological risk assessment was estimated by using the following equation given by Hakanson (1980):

$$({}^iEr) ={}^iT_r \times {}^iC_f = {}^iT_r \times ({}^iC_o/{}^iC_n);$$

$$\text{RI}=\sum_{i=1}^{7}{}^iEr=\sum_{i=7}^{7}{}^iTr\times {}^iCf$$

where ^*i*^*E*_*r*_ is the ecological risk factor of metal “*i*”; ^*i*^*T*_*r*_ and ^*i*^*C*_*f*_ are the toxic response factor and contamination factor for metal “*i*,” respectively; and ^*i*^*C*_*o*_ and ^*i*^*C*_*n*_ are the concentrations measured for the metal “*i*” in the sediment sample and its reference value. The values of ^*i*^*T*_*r*_ are 30, 5, 5, 2, 10, and 1 for metals Cd, Cu, Ni, Pb, Cr, As, and Zn, respectively. Further, sediments were assessed for potential ecological risk using the standard values (Table 5) given by Hakanson (1980).

## Results and discussion

### Sediment characteristics and organic carbon

Descriptive statistics of various sediment components are given in Table 1 and downcore profiles of core A and core B are discussed separately in the following section.

**Table 1:** Descriptive statistics of various sediment components and trace metals (µg/g)

	**Core A (u/s)**				**Core B (d/s)**			
	**Min**	**Max**	**Mean**	**SD**	**Min**	**Max**	**Mean**	**SD**
Sand (%)	53.74	89.58	75.79	9.77	46.58	95.52	84.94	8.54
Silt (%)	8.20	40.99	20.14	8.52	0.31	40.47	9.69	7.07
Clay (%)	0.25	8.73	4.07	1.88	0.04	11.27	2.21	2.05
OC (%)	0.61	1.84	0.97	0.32	0.05	2.66	0.54	0.59
As	6.87	19.86	14.13	3.77	4.65	14.90	8.31	1.97
Cd	0.00	0.30	0.06	0.07	0.01	0.27	0.09	0.07
Co	4.42	19.69	11.45	3.78	3.81	15.85	5.77	2.51
**Cr**	20.35	82.26	51.62	14.30	14.03	65.27	21.12	10.13
Cu	0.70	15.58	6.58	4.05	0.23	65.85	11.38	13.04
Ni	8.98	38.43	22.00	7.18	5.44	30.51	8.38	4.96
Pb	13.61	55.52	30.11	10.19	1.24	29.89	8.51	6.34
Zn	32.57	380.37	193.38	124.00	21.77	158.52	68.08	34.25
Al	2.10	10.27	4.24	1.92	1.58	3.75	2.12	0.47
Fe	0.67	2.33	1.48	0.41	0.43	1.70	0.62	0.26
Mn	0.00	0.02	0.01	0.00	0.00	0.01	0.01	0.00

*u/s* upstream estuary and *d/s* downstream estuary

#### Core A:

**Fig. 2:**
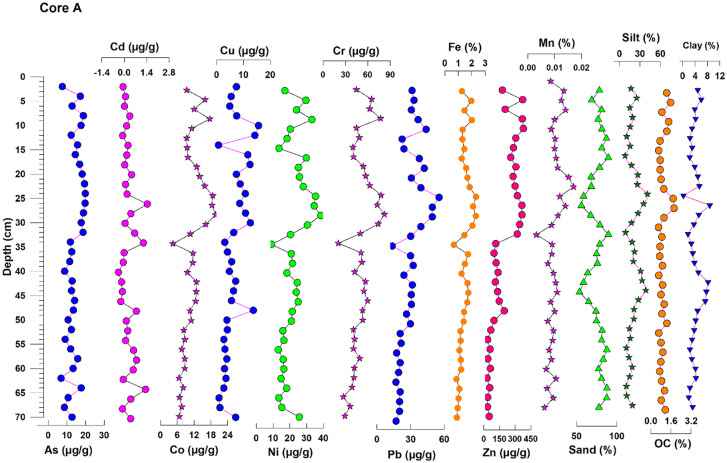
Down core variations of sediment components and trace metals in core A (upstream) estuary

The sand percentage varied from 53.74 to 89.58%, with a mean (± s.d.) value of 75.79% (± 9.77%) in the upper estuary. Higher sand content was found in the core’s surface layers (between 8 and 18 cm) and the bottom layers (between 52 and 70 cm), indicating that a relatively high hydrodynamic energy state existed, allowing coarser fractions to deposit more quickly. Sand content followed a decreasing trend and reached a minimum value of 53.74% at 26 cm (Fig. 2), balanced by the higher silt percentage (40.99%).

Similar behavior was noted at the layer 44 cm; here silt was higher and sand content was low, which indicates a calmer environment with the low hydrodynamic condition. Overall, the sand distribution showed cyclical periods of “low hydrodynamic conditions” between slices 4, 26, 44, and 58 subsections of the sediments.

**Fig. 3:**
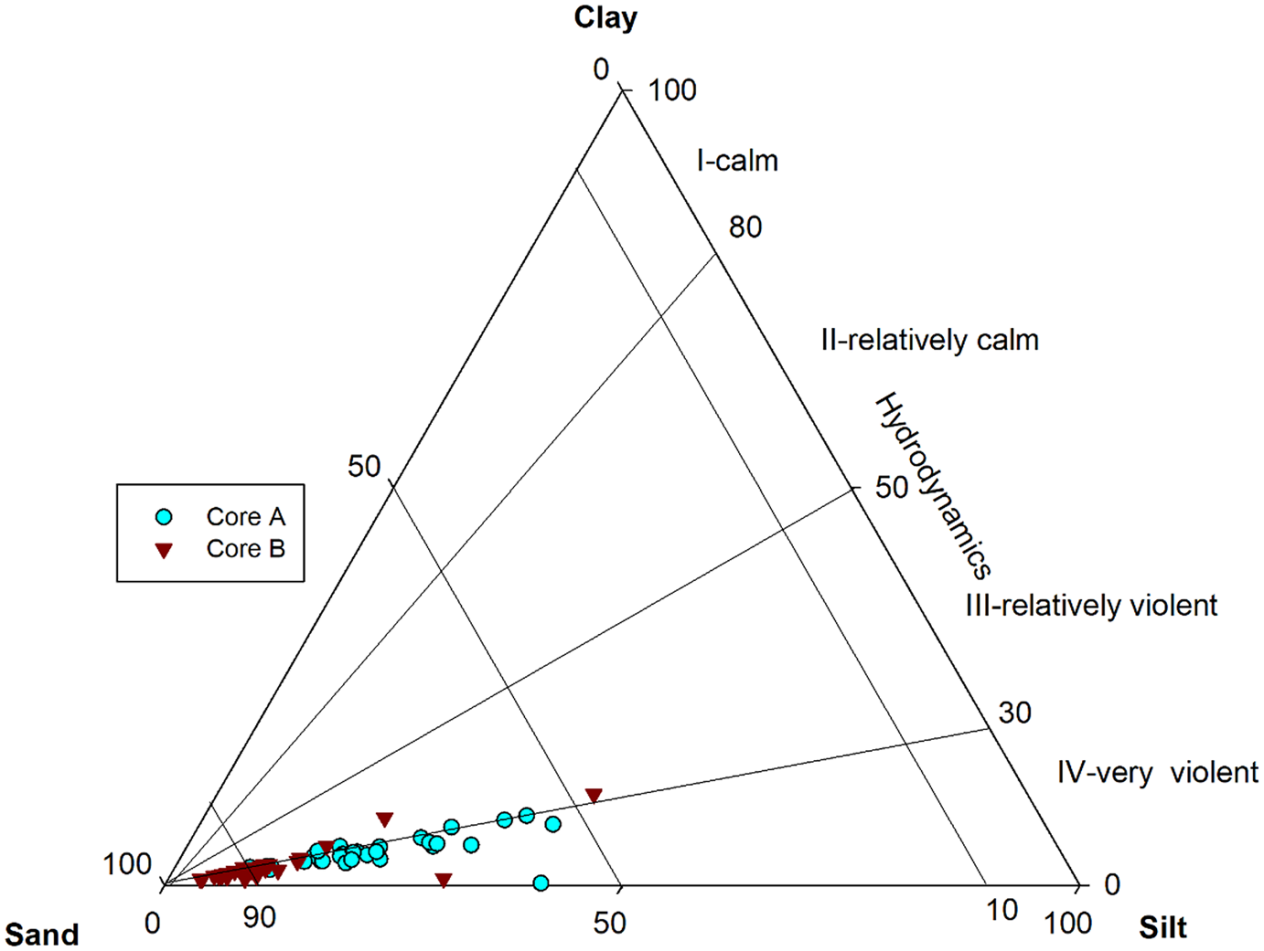
Ternary plot to classify various hydrodynamic conditions after Perjup (1988)

Figure 3 is a ternary plot displaying the per cent data of sand, silt, and clay that helps understand the particle size distribution in the depositional environment (Perjup, 1988). From Fig. 3, it is revealed that their deposition took place in relatively violent to very violent hydrodynamic energy conditions.

The quantity of silt varied from 8.2 to 40.9%, with a mean (± s.d.) value of 20% (± 8.52). At depths of 24 cm and 42 cm, the silt percentage reached the maximum value (40.99%). Percent clay varied from 0.25 to 8.8%, with a mean (± s.d.) value of 4.07% (± 1.88). Similarly, organic carbon ranged from 0.61 to 1.84%, with a mean value of 0.97% (± 0.32). It showed a good association with the silt, which is bolstered by the positive correlation between silt and organic carbon (*r* = 0.6, *p* < 0.01) and negative correlation with sand (*r* =  − 0.7, *p* < 0.01).

Thus, comparatively higher values of silt and clay were observed for the middle half of the core, indicating calm and less turbulent with low-energy conditions, which facilitated the deposition of finer sediments. Hence from the results, it is clear that higher organic carbon values were observed wherever silt was high, and sand was low. The concentration of organic carbon decreased with depth in both the cores due to degradation. This is because oxygenated water percolates more quickly into coarser particles than finer particles, resulting in a faster degradation rate (Dessai & Nayak, 2009; Fernandes & Nayak, 2017, 2020).

#### Core B:

**Fig. 4:**
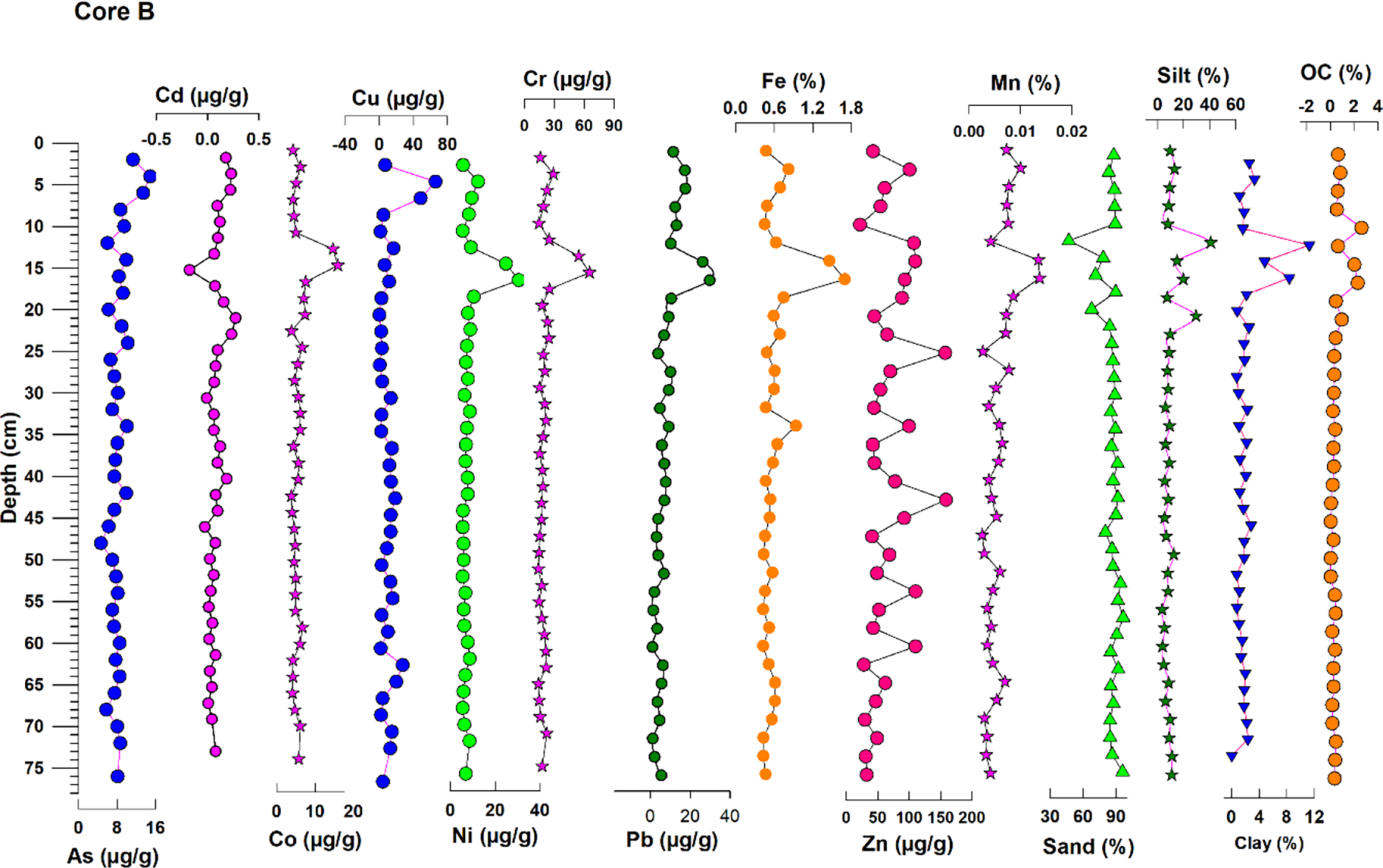
Down core variations of sediment components and trace metals in core B (downstream)

The sand ranges from 46.58 to 95.52%, with a mean (± s.d.) value of 84.94% (± 8.54) (Fig. 4). Sand shows a sudden decline between the layers (11 to 17 cm), which is well balanced by the increase in silt, organic carbon, and clay content in these layers. It shows a sharp increase after these layers and remains constant throughout the section. The silt ranges from 0.31 to 40.47%, with a mean (± s.d.) value of 9.8% (± 7.07) and exhibits the opposite trend compared to the sand’s profile. Furthermore, clay also shows the opposite trend with the sand and ranges from 0.04 to 11.27%, with a mean (± s.d.) value of 2.2% (± 7.07). Organic carbon profile matched well with the profiles of silt and clay. Increased surface area available in the silt and clay on fine grain size might have controlled organic carbon accumulation (Dessai et al., 2009; Liu et al., 2016).

Overall, core A showed a high percentage of finer fractions and organic carbon (nearly two-fold) than core B. This is mainly because of the presence of mangroves near the core A’s location. In both cores, organic carbon showed a decline with depth because of their degradation (Fernandes and Nayak, 2020).

### Geochemistry of metals

The geochemical data for sediment core A and core B (Table 1) and down core profiles (Figs. 2 and 4) are discussed in this section.

#### Major elements (Fe, Al, and Mn)

In core A, the Fe concentration varied from 0.67 to 2.33% (mean = 1.46%), and in core B (downstream estuary) between 0.43% and 1.70% (mean = 0.62%). Similarly, Al varied from 2.10 to 10.27% (mean = 4.22%), and in core B between 1.58% and 3.75% (mean = 2.12%). In comparison, Mn did not vary much, with a mean value of 0.01% in both the cores. A similar distribution pattern is found for Mn and Fe in cores A and B, and they showed perfect association with the rest of the elements and sediment components. A strong positive correlation between Fe and Mn (*r* = 0.8, *p* < 0.01) indicates their common source of origin in both the cores. This may be attributed to the form of oxyhydroxides (Fernandes & Nayak, 2015). The verticle profile of Fe follows the profile of silt up to 36-cm depth in core A and between 12 and 18 cm in core B.

Further down-core, the concentration remains constant in both the cores. From the literature, Mn remains predominantly in the solution phase up to salinity 10 PSU and eventually undergoes flocculation accompanied (Dessai et al., 2009; Kerdijk & Salomons, 1981) by precipitation at salinity 18 PSU. At the same time Fe gets precipitated at very low salinity. Similar behavior was observed in the present study for Fe and Mn. As river input was less in the pre-monsoon and salinity was very high, it favored the precipitation of Mn at a salinity of ~ 22 PSU. In general, both Fe and Mn precipitate as oxides and hydroxides in the top layers of the sediments, whereas minimal accumulation takes place in the bottom layers due to dissolution and migration. This could be because of oxic conditions at the surface and suboxic to anoxic conditions down-core (Klinkhammer et al, 1982; Santschi et al., 1990; Fernandes and Nayak, 2020).

#### Trace elements (As, Cd, Ni, Co, Cu, Cr, Zn, and Pb)

In core A (Fig. 2), the elements, viz., Co, Cu, Ni, As, Cr, and Pb, showed a similar trend as silt, organic carbon, and clay in layers between 2 and 10 cm and 12 to 34 cm. After 34 cm, almost all the elements showed no significant variations which is mainly because of the increase in sand content followed by decline in the silt content. Whereas, the element Cd remained constant in the surface layers and showed variations in the middle slices of the core, i.e., between 26 and 38 cm. Wherever mud fraction and organic carbon were high, trace element concentration also increased, showing the association of trace elements with the finer sediments in the form of organometallic complexes (Fernandes & Nayak, 2015). The inference is also supported by the correlation coefficient analysis (Table 2). Other than Cd, all the trace elements show a strong positive correlation (> 0.8) with Fe and Mn and moderate correlation with Al (> 0.5). They exhibit a negative correlation with sand and good positive relation with silt and organic carbon. The strong relationship of the trace metals with Fe and Mn indicates their excellent scavenging capacity. Similarly, in core B (Fig. 4), Co, Ni, Cr, and Pb showed a similar trend in layers between 12 and 18 cm. After 18 cm, almost all the elements showed no significant variations except Zn which showed slight variations towards the depth. This behavior was precisely matching the profile of silt, clay, and organic carbon, which is supported by correlation coefficient analysis (Table 2). This is also supported by the results obtained from the correlation regression analysis. Majority of the trace elements show a strong positive correlation (> 0.8) with Fe and Mn and moderate correlation with Al (> 0.5) except As, Cd, Cu, and Zn. They showed a negative correlation with sand and good positive relation with clay and organic carbon. Metals would thus be correlated with organic matter and finer fractions during the initial deposition. In contrast, metals redistribute as organic matter degrades with depth, resulting in a gradual decrease in metal concentration (Allen & Duffy, 1998).

**Table 2:** Correlation between sediment components and trace metals in core A and core B from Sita-Swarna estuary

	**Sand**	**Silt**	**Clay**	**OC**	**As**	**Cd**	**Co**	**Cr**	**Cu**	**Ni**	**Pb**	**Zn**	**Al**	**Fe**	**Mn**
**Core A**															
Sand	1														
Silt	− 0.988**	1													
Clay	− 0.721**	0.607**	1												
OC	− 0.409*	0.426*	0.198	1											
As	− 0.200	0.225	0.021	0.369*	1										
Cd	− 0.247	0.200	0.356	0.402	0.343	1									
Co	− 0.617**	0.623**	0.385*	0.547**	0.710**	0.094	1								
Cr	− 0.549**	0.549**	0.364*	0.502**	0.628**	0.042	0.958**	1							
Cu	− 0.145	0.152	0.066	0.168	0.454**	0.011	0.506**	0.492**	1						
Ni	− 0.531**	0.535**	0.335	0.517**	0.678**	0.126	0.948**	0.911**	0.576**	1					
Pb	− 0.486**	0.513**	0.203	0.561**	0.665**	0.105	0.877**	0.791**	0.667**	0.886**	1				
Zn	− 0.212	0.221	0.101	0.389*	0.745**	0.049	0.743**	0.660**	0.708**	0.719**	0.766**	1			
Al	− 0.051	0.075	− 0.077	0.285	0.558**	− 0.010	0.442**	0.314	0.660**	0.549**	0.738**	0.615**	1		
Fe	− 0.627**	0.638**	0.367*	0.471**	0.687**	0.048	0.978**	0.940**	0.472**	0.911**	0.844**	0.706**	0.372*	1	
Mn	− 0.422*	0.454**	0.136	0.586**	0.666**	− 0.087	0.859**	0.831**	0.351*	0.824**	0.762**	0.619**	0.458**	0.832**	1
**Core B**															
Sand	1														
Silt	− 0.981**	1													
Clay	− 0.842**	0.781**	1												
OC	− 0.318	0.334*	0.423**	1											
As	0.178	− 0.138	− 0.039	0.256	1										
Cd	− 0.137	0.179	0.092	0.166	0.551**	1									
Co	− 0.325*	0.281	0.476**	0.629**	0.144	− 0.007	1								
Cr	− 0.408*	0.355*	0.621**	0.633**	0.269	0.126	0.926**	1							
Cu	− 0.034	0.069	0.124	− 0.046	0.613**	0.317	− 0.042	0.128	1						
Ni	− 0.381*	0.339*	0.591**	0.659**	0.250	0.118	0.947**	0.986**	0.129	1					
Pb	− 0.369*	0.366*	0.536**	0.758**	0.410*	0.282	0.781**	0.831**	0.220	0.856**	1				
Zn	− 0.169	0.139	0.292	0.019	0.120	0.334	0.253	0.320	0.036	0.315	0.262	1			
Al	− 0.197	0.204	0.396*	0.655**	0.611**	0.310	0.707**	0.794**	0.451**	0.810**	0.915**	0.134	1		
Fe	− 0.369*	0.318	0.557**	0.614**	0.226	0.073	0.939**	0.955**	0.105	0.963**	0.843**	0.281	0.782**	1	
Mn	− 0.247	0.242	0.374*	0.708**	0.428**	0.269	0.839**	0.779**	0.130	0.815**	0.896**	0.134	0.828**	0.828**	1

**Correlation is significant at the 0.01 level (2-tailed). *Correlation is significant at the 0.05 level (2-tailed)

#### Principal component/factor analysis

Principal component analysis (PCA) performed for the data sets explained three principal components (PCs) for core A and three PCs for core B with a total variance of 74% and 71%, respectively (Table 3). The experimental data was standardized initially to avoid misclassification because of the wide range in the data set. Further, sampling adequacy and the suitability of the data for performing PCA and FA were checked by Kaiser and Bartlett’s sphericity tests. A high value of Kaiser–Meyer–Olkin (KMO), close to 1, indicated the sampling adequacy, and the criteria were met by the current data set (> 0.86). Similarly, we checked the correlation matrix for the dataset and found a significance level of 0, indicating a significant relationship between the variables. The PCA data helped to understand the metal distribution pattern and categorize the variables with important significance.

**Table 3:** Principal component loadings of trace metals and sediment properties for the Sita-Swarna estuary

Variables	**Core A**			**Core B**			
	PC1 (46.5%)	PC 2 (19.7%)	PC 3 (9.7%)	PC1 (37.6%)	PC 2 (16.6%)	PC 3 (14.0%)	PC 4 (8.0%)
Sand	− 0.127	** − 0.944**	0.020	− 0.193	** − 0.920**	− 0.111	0.052
Silt	0.100	**0.912**	− 0.053	0.121	**0.906**	0.163	− 0.085
Clay	0.191	**0.646**	0.148	0.195	**0.909**	0.051	0.013
OC	− 0.076	**0.756**	0.038	− 0.008	0.180	**0.826**	− 0.058
As	0.603	− 0.198	**0.687**	− 0.134	− 0.353	− 0.255	0.479
Cd	− 0.077	0.113	**0.969**	− 0.037	− 0.135	0.179	**0.827**
Co	**0.918**	0.377	0.021	**0.919**	− 0.054	0.222	− 0.066
Cr	**0.964**	0.127	0.071	**0.839**	0.375	− 0.032	0.159
Cu	0.224	0.389	− 0.029	− 0.334	0.290	− 0.360	− 0.046
Ni	**0.866**	0.384	0.039	**0.857**	0.326	0.188	0.051
Pb	**0.797**	0.496	− 0.012	0.144	0.398	**0.771**	− 0.049
Zn	0.320	0.510	− 0.051	0.295	0.286	− 0.262	0.7**26**
Fe	**0.965**	0.147	0.116	**0.946**	0.202	0.129	− 0.025
Mn	**0.877**	− 0.070	− 0.020	0.471	− 0.097	**0.749**	− 0.046

**Fig. 5:**
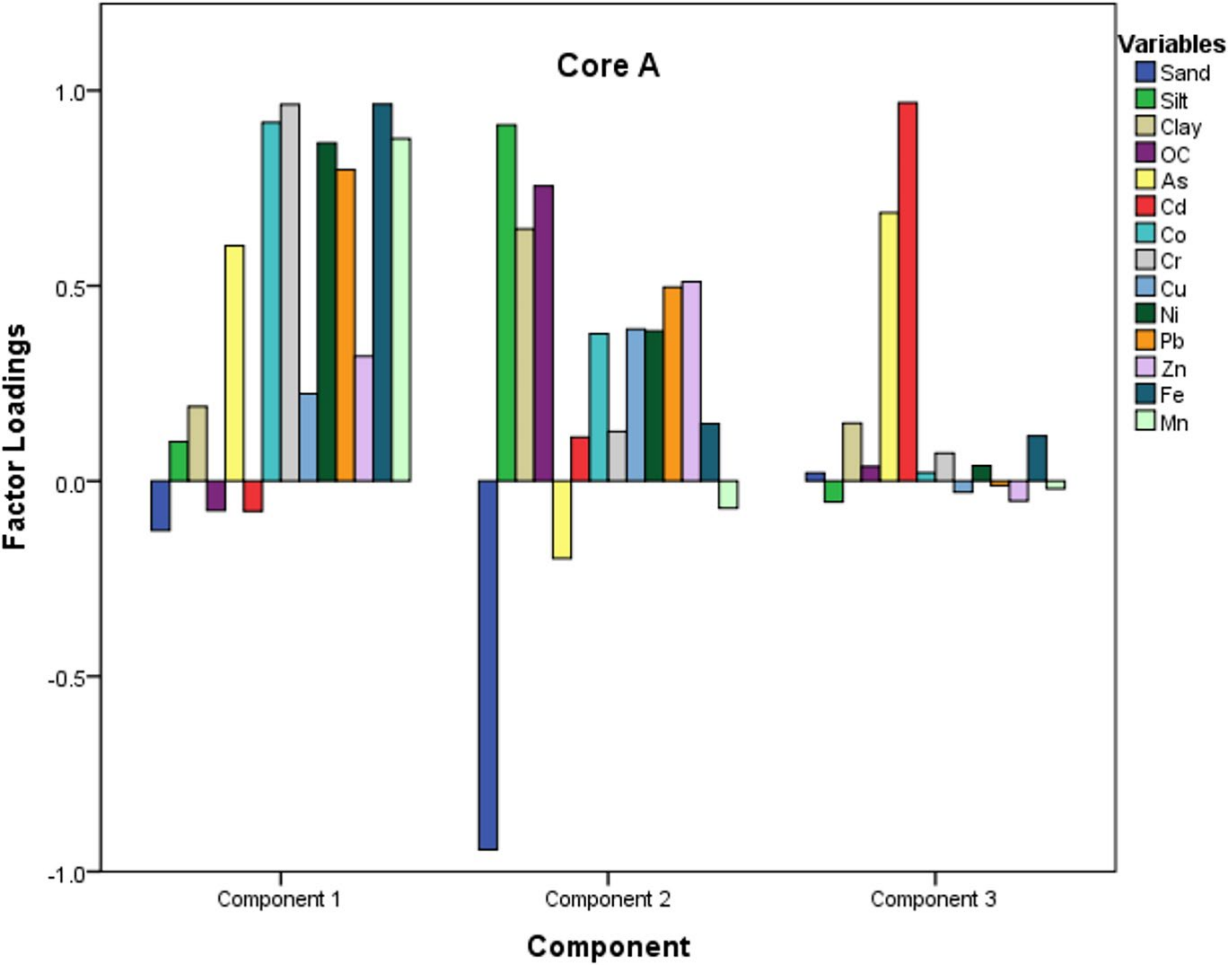
Loading plot for core A raised in Swarna estuary (upstream)

**Fig. 6:**
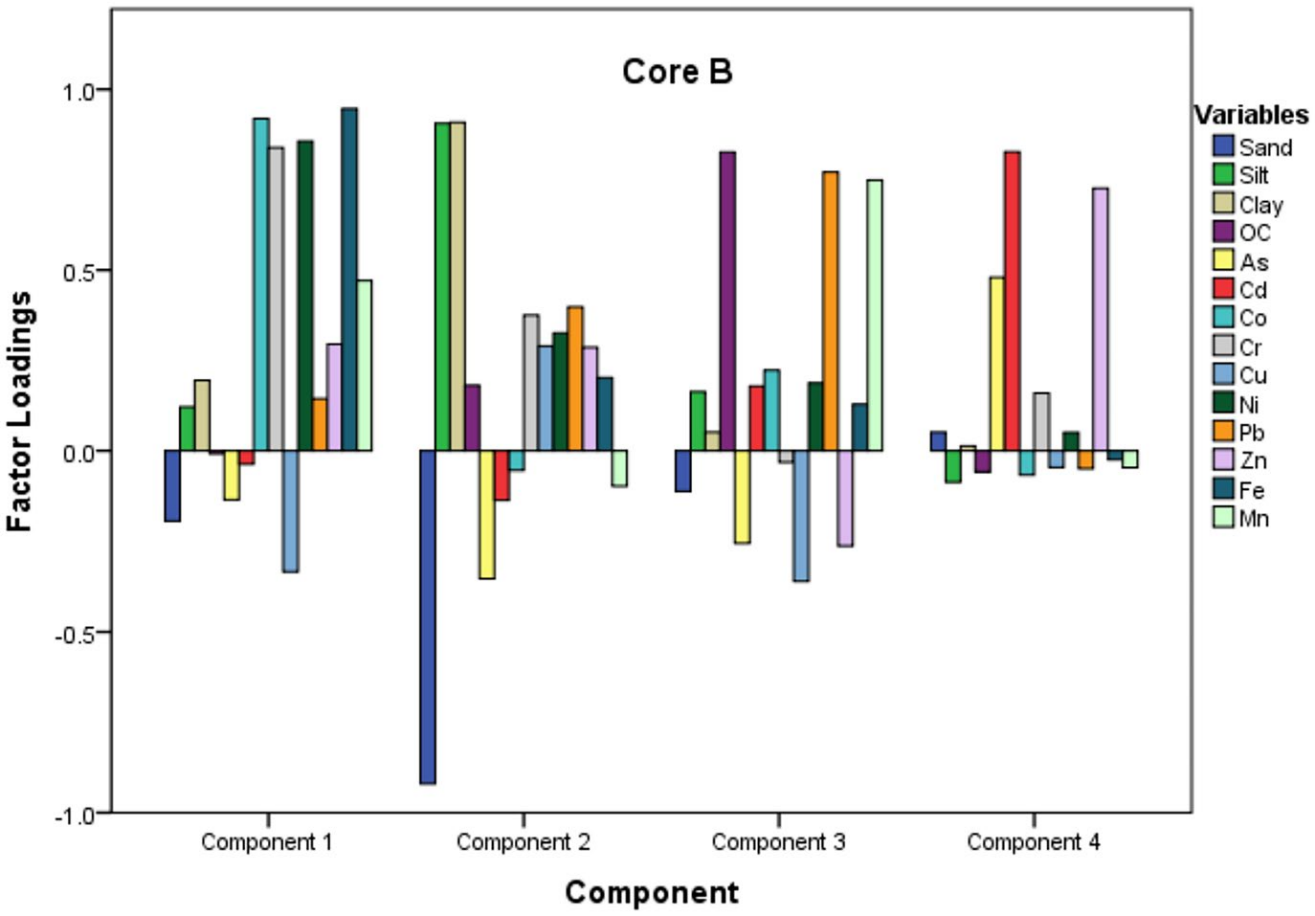
Loading plot for core B raised in Swarna estuary (downstream)

Furthermore, to provide a simple visualization of the data (Figs. 5 and 6), PCA is described via loadings and score plots. The first PC in core A accounted for 46.5% and showed high positive loadings > 0.7) for elements such as Co, Cr, Ni, Pb, Fe, and Mn. Metals such as Zn, Cd, and Cu showed very weak loadings in these components. We have named this factor as the “Fe–Mn oxide controlled factor.” Thus both natural and anthropogenic sources may be attributed to PC1. The second PC accounted for 19.7% of the total variance and showed a strong association of silt, clay, and OC with high positive loading (> 0.7). This factor may be called an “organic matter controlled factor.” In contrast, sand showed strong negative loading (− 0.9), with all the other elements showing very weak loadings to sediment properties. Hence, we can conclude that only natural sources control the variations in PC2. The third component showed the most negligible variance and loaded with only two variables, As and Cd (> 0.6), which could be because of the anthropogenic sources.

In core B (Fig. 6), PC1 accounted for 37.6%, followed by high positive loadings (> 0.7) for elements such as Co, Cr, Ni, and Fe. Whereas Cu, Zn, and Pb showed no association with all other metals, and their weak loading in PC1 indicates a different source of origin.

Additionally, PC2 (16.6% variance) and PC3 (14.0% variance) have strong positive loadings for clay, silt, and OC and Mn, respectively, by representing the pure lithogenic origin. Strong positive loadings dominated PC4 (8.0%) with Zn and Cd. Natural and anthropogenic sources may be attributed to variations in PC1 and PC4 in core B, while natural sources purely control PC2 and PC3.

## Quality assessment of the sediments

### Enrichment factor (EF)

**Fig. 7:**
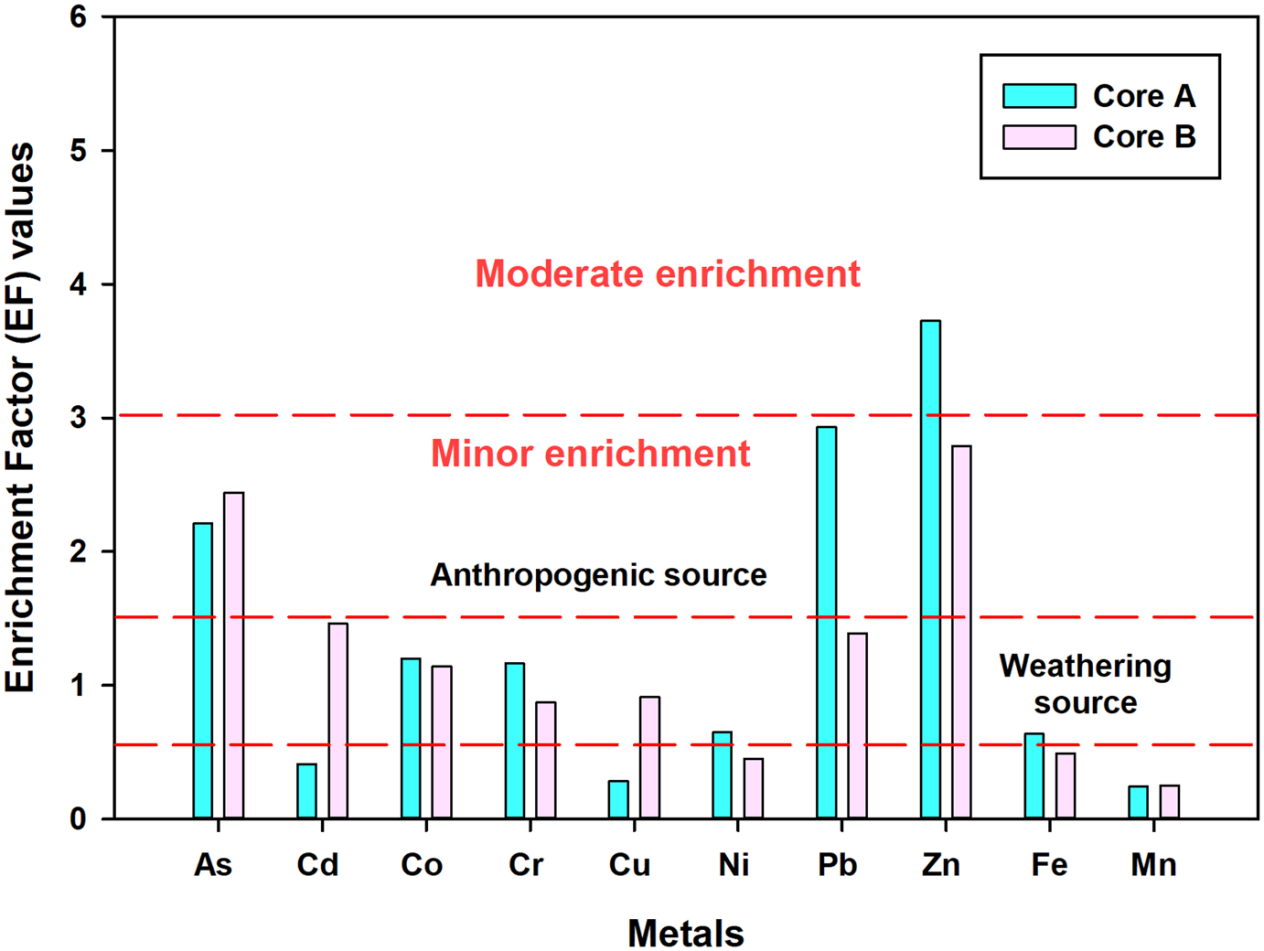
Normalized EF values in the sediments of Sita-Swarna estuary

According to Ergin et al. (1991), enrichment values between 0.5 and 1.5 indicate the natural origin, and values > 1.5 indicate anthropogenic origin. Based on the EF values obtained for the present study, results were interpreted using the standard values given in Table 4. The EF values estimated for the dataset followed the order of Zn > Pb > As > Co > Cr > Ni > Fe > Cd > Cu > Mn. In core A, we found comparatively higher EF values vis-a-vis core B (Fig. 7), primarily due to the high percentage of finer fractions. Mean concentrations of Co, Cr, Ni, Pb, Zn, and Fe were higher in core A than core B for the world average shale. Closeness to urban areas, harbor, movement of boats, discharge of municipal waste, and surface runoff are the primary sources of these metals. There was negligible enrichment in the EF values for Fe, Mn, Ni, and Cu, whereas As, Co, Pb, Cr, Cd, and Zn showed mild to moderate enrichment. Hence, continuous pollution monitoring of the marine water bodies and sediment quality will help us to know the present contamination level. Also, suitable preventive measures can be taken to prevent further damage the sedimentary environment.

**Table 4:** Enrichment factor (Ergin et al., 1991) and *I*_geo_ (Muller, 1969) values to assess sediment quality

EF classes	Sediment quality	*I*_geo_	*I*_geo_ class	Sediment quality
EF < 1	No enrichment	0–0	0	Unpolluted
EF < 3	Minor enrichment	0–1	1	Unpolluted to moderately polluted
EF 3–5	Moderate enrichment	1–2	2	Moderately polluted
EF 5–10	Moderately severe enrichment	2–3	3	Moderately to highly polluted
EF 10–25	Severe enrichment	3–4	4	Highly polluted
EF 25–50	Extremely severe enrichment	4–5	5	Highly to very highly polluted
		5–6	> 5	Very highly polluted

### Geoaccumulation index (I_geo_)

**Fig. 8:**
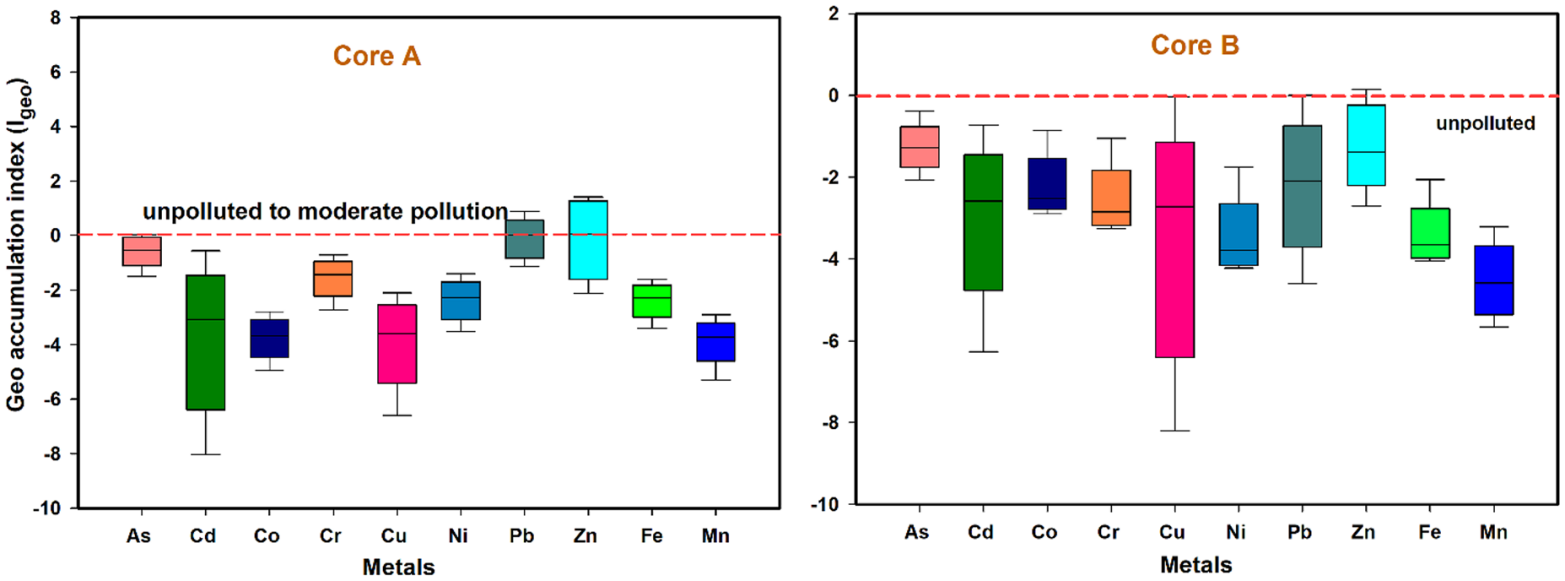
Box plots representing the *I*_geo_ values in the sediments of Sita-Swarna estuary

*I*_geo_ values estimated for Zn, As, Pb, Co, Cu, Cr, Fe, Ni, Cd, and Mn were less than zero, suggesting that the site is not polluted with these metals (Fig. 8).

### Potential ecological risk

In the current study, the risk index (RI) proposed by Hakanson (1980) was used to assess the potential ecological risk on the aquatic environment. The values of ecological risk coefficients (^*i*^*E*_*r*_) for As, Cd, Cr, Cu, Ni, Pb, and Zn are presented in Table 5.

**Table 5:** Potential ecological risk indices and toxicity response indices based on Hakanson (1980)

(^*i*^*E*_*r*_) and RI based on Hakanson (1980)			Potential ecological risk indices for individual metal (^*i*^*E*_*r*)_ — for the current study						
**Potential (**^***i***^***E***_***r***_**)**	**Toxicity (RI)**	**Ecological risk level**	**Elements**	**Core A**			**Core B**		
				**Mean**	**Min**	**Max**	**Mean**	**Min**	**Max**
^*i*^*E*_*r*_ < 40	RI < 150	Low risk	As	10.84	5.29	15.28	6.40	3.57	11.46
40 < ^*i*^*E*_*r*_ < 80	150 < RI < 300	Moderate risk	Cd	6.43	0.17	30.43	9.43	0.59	27.21
80 < ^*i*^*E*_*r*_ < 160	300 < RI < 600	Considerable risk	Cr	1.13	0.45	1.83	0.47	0.31	1.45
160 < ^*i*^*E*_*r*_ < 320		High risk	Cu	0.73	0.08	1.73	1.26	0.03	7.32
^*i*^*E*_*r*_ < 320	600 < RI	Very high risk	Ni	1.63	0.66	2.83	0.62	0.40	2.24
			Pb	7.43	3.40	13.88	2.01	0.03	7.47
			Zn	1.99	0.34	4.00	0.72	0.23	1.67
			**(RI)**	**30.19**	**10.39**	**69.98**	**20.90**	**5.16**	**58.82**

The values of ^*i*^*E*_*r*_ were lower than 40 for all the elements analyzed in the study. And, the values of RI were lower than 150 in both the cores A and B indicating low risk for the aquatic life. These results are also in good agreement with the values of *I*_geo_, indicating less pollution in the catchment.

## Conclusion

From the above study, the following conclusions can be drawn:

Core A showed a high concentration of metals than core B because of the surrounding mangroves near core A. Further, the amount of organic carbon and percentage of finer fractions was approximately two-fold higher near mangroves, highlighting the efficiency of mangroves in sequestering the trace metals. The PCA data explained three PCs for core A and three PCs for core B with a total variance of 76% and 77%, respectively. The study revealed that Fe–Mn oxyhydroxides and organic carbon are the two crucial factors that controlled metals’ distribution in the catchment. Further, based on the *I*_geo_ and enrichment values, the site can be categorized to show less pollution with all the metals analyzed. The *I*_geo_ values for Pb and Zn were slightly higher in both the cores and indicate moderate pollution in the catchment. The potential ecological risk index (^*i*^*E*_*r*_) for all metals was found to be less than 40, and the toxicity response index (RI) was below 150, indicating a no risk to aquatic life in the catchment. Hence the present study can be used as baseline data to monitor and protect the fragile coastal environment.

## Data Availability

The datasets generated during and/or analyzed during the current study are available from the corresponding author on reasonable request.
